# The Identification of Proteolytic Substrates of Calpain-5 with N-Terminomics

**DOI:** 10.3390/ijms26136459

**Published:** 2025-07-04

**Authors:** Jozsef Gal, Antoine Dufour, Daniel Young, Eddy S. Yang, James W. Geddes

**Affiliations:** 1 Spinal Cord and Brain Injury Research Center (SCoBIRC), University of Kentucky, Lexington, KY 40536, USA; 2 Department of Neuroscience, University of Kentucky, Lexington, KY 40536, USA; 3 Department of Radiation Medicine, University of Kentucky, Lexington, KY 40536, USA; 4 Markey Cancer Center, University of Kentucky, Lexington, KY 40536, USA; 5 Department of Physiology and Pharmacology, Cumming School of Medicine, University of Calgary, Calgary, AB T2N 1N4, Canada; 6 Snyder Institute for Chronic Diseases, Cumming School of Medicine, University of Calgary, Calgary, AB T2N 1N4, Canada

**Keywords:** CAPN5, protease, N-terminomics, TAILS, disease

## Abstract

Calpain-5/CAPN5 is a calcium-activated, non-lysosomal cysteine (thiol) protease. The substrate repertoire of CAPN5 is not known. Calpains catalyze limited proteolysis of their substrates, generating neo-N-termini that correspond to internal residues of their nascent substrate proteins. To identify such neo-N-termini generated by CAPN5, we employed an N-terminomics approach called TAILS (Terminal amine isotopic labeling of substrates) to quantitatively compare the N-terminal peptides detected in parental and *CAPN5*-deficient SH-SY5Y neuroblastoma cells. Thirty neo-N-termini corresponding to 29 protein groups and 24 unique proteins were detected to be depleted in the *CAPN5^−/−^* cells. A subset of the identified putative substrates was further studied with CAPN5 co-immunoprecipitation, in vitro calcium-induced CAPN5 proteolysis assay, and their cellular fragmentation patterns were compared in parental and *CAPN5*-deficient SH-SY5Y cells. Here, we provide evidence for CAPN5-mediated proteolysis of the synaptic proteins DLGAP4, IQSEC1 and MPDZ, the neurodegeneration-related EWS, hnRNPU, TFG and UGP2, the DNA replication regulator MCM3, and the neuronal differentiation regulator LMTK1. Our data provide new relevance for neovascular inflammatory vitreoretinopathy (NIV), a progressive eye disease caused by pathogenic mutations in CAPN5. Data are available via ProteomeXchange with identifier PXD064313.

## 1. Introduction

Calpains are calcium-dependent, non-lysosomal proteases with a critical cysteine residue in their active center. Calpains regulate cellular processes via limited proteolysis of their substrate proteins [1,2,3,4]. Calpains are defined by the presence of the conserved calpain catalytic domain. The human genome encodes 15 calpains. Calpain-5/CAPN5 is categorized as a non-classical calpain because it contains a C-terminal calcium-binding C2 domain instead of the penta-EF hand domain of classical calpains [5,6]. The *CAPN5* mRNA is one of the highest expressed calpain mRNAs in the central nervous system (CNS) of rats [7], and is also highly expressed in other tissues including the gastrointestinal tract, kidney, liver, testis and trachea [5]. The CAPN5 protein is expressed at relatively high levels in synapses and in the outer segment of photoreceptor cells [8]. At the subcellular level, CAPN5 was detected in the plasma membrane, cytosol, mitochondria and nuclei [6,7,8,9,10,11]. The C2 domain was shown to mediate the membrane association of CAPN5 [6] which is further stabilized by cysteine S-acylation [10]. CAPN5 appeared to require membrane association for full catalytic activity [6,10,11].

Mutations in CAPN5 cause the devastating eye disease neovascular inflammatory vitreoretinopathy (NIV) [11,12,13,14,15]. The NIV-related mutations in CAPN5 cause altered enzyme localization, S-acylation and activity [11,12,13,14,15].

To better understand the physiological roles of CAPN5 and the pathological mechanisms mediated by CAPN5, it is important to identify its substrates. However, the mechanisms involved in the substrate recognition of calpains are incompletely understood with no apparent consensus sequences [16,17,18,19]. Previously identified CAPN5 substrates include SLIT2 [20], Caspase-4 [21], the autoimmune regulator AIRE [11] and CAPN5 itself is an autoproteolytic substrate [6,10]. We recently identified additional candidate interaction partners of CAPN5 with a proteomic screen based on co-immunoprecipitation. Several interaction partners were also in vitro substrates of CAPN5 (Gal et al., manuscript in preparation).

Our goal in this study was to identify novel CAPN5 substrates in the CNS. The SH-SY5Y human neuroblastoma cells are one of the most widely used and best characterized human cell lines for research on neuronal cell biology. This cell line expresses *CAPN5* mRNA at relatively high levels. The cell line exhibits neuron-like enzyme activity and expresses neuron-like receptors [22,23,24]. Our previous studies have employed this cell line for studies of CAPN5 [6,7,10,11].

Here we describe the identification of further candidate CAPN5 substrates with the Terminal amine isotopic labeling of substrates (TAILS) N-terminomics method [25,26]. Proteases generate novel protein N-termini, so-called neo-N-termini, that correspond to internal residues of their nascent substrates. The TAILS method identifies and quantitatively compares the levels of N-terminal peptides in two samples. We used TAILS to compare the “N-terminomes” of parental and *CAPN5*-deficient (*CAPN5^−/−^*) SH-SY5Y cells. We identified 30 neo-N-termini attributable to proteolysis by CAPN5, representing 24 unique proteins and five groups of closely related proteins. We further characterized nine CAPN5 proteolytic substrate candidates with co-immunoprecipitation and in vitro CAPN5 protease assay and compared their cellular fragmentation in parental and *CAPN5^−/−^* SH-SY5Y cells. We discuss the potential relevance of our findings to the etiology of human diseases including NIV.

## 2. Results

### 2.1. Inactivation of CAPN5 in the SH-SY5Y Cell Line

**Figure 1:**
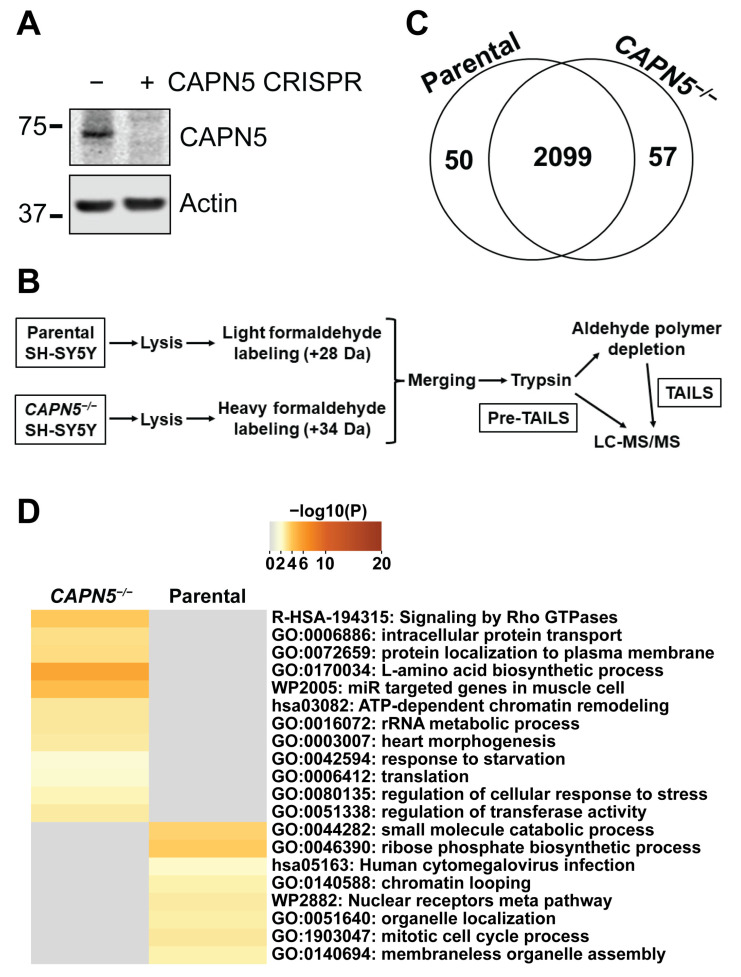
The effect of the inactivation of *CAPN5* on the proteome of SH-SY5Y cells. (**A**) Immunoblotting showing the inactivation of *CAPN5* with CRISPR technology. The bars indicate molecular weight marker bands (kDa). (**B**) Schematic of our proteomics workflow. (**C**) Most proteins detected by the pre-TAILS shotgun proteomics were identified with peptides that were at similar levels in the parental and the *CAPN5^−/−^* SH-SY5Y cells. A small number of proteins were identified only by peptides at significantly higher levels in the parental or the *CAPN5^−/−^* cells. (**D**) Pathway analysis of the differentially expressed proteins by Metascape.

Following inactivation of CAPN5 using the Double Nickase CRISPR method [27], cells were subjected to single-cell cloning and a clone with no detectable CAPN5 protein was chosen for further studies (Figure 1A).

### 2.2. Comparison of the Total Proteomes of Parental and CAPN5^−/−^ SH-SY5Y Cells

Our proteomics workflow, based on three biological replicates, is summarized in Figure 1B. Formaldehyde reacts with the primary amine groups of lysine residues and unblocked N-termini. The proteins from the parental SH-SY5Y cells were labeled with light formaldehyde (adds 28 Da), whereas the proteins from the *CAPN5^−/−^* SH-SY5Y cells were labeled with heavy/deuterated formaldehyde (adds 34 Da). After merging the formaldehyde-labeled lysates, the proteins were subjected to trypsin digestion.

To determine the effect of the CAPN5 loss on the total proteome, the tryptic peptide digest was subjected to shotgun LC-MS/MS proteomics (this is the “pre-TAILS” sample). The 6 Da differential mass labeling enables quantitative comparison of the abundances of the detected peptides derived from the parental and the *CAPN5^−/−^* SH-SY5Y cells. An interquartile boxplot analysis [28] was applied to determine whether the identified peptides were at significantly different levels in the two cell lines.

After the removal of keratins and other known contaminants, 2099 unique proteins were at comparable levels in the parental and the *CAPN5^−/−^* SH-SY5Y cells (Figure 1C, Tables S1 and S2). Fifty proteins were identified only with peptides that were significantly depleted in the *CAPN5^−/−^* SH-SY5Y cells, whereas 57 proteins were identified only with peptides that were at significantly higher levels in the *CAPN5^−/−^* SH-SY5Y cells.

We subjected the genes of the proteins that were identified by peptides at altered levels to pathway and annotation term analysis by the Metascape website tool [29] (Figure 1D). One of the proteins that was expressed higher in the parental SH-SY5Y cells, FLJ45252 (UniProt: YJ005_HUMAN), an uncharacterized protein, was excluded from the Metascape analysis. The results showed that although the inactivation of *CAPN5* caused only limited changes in the proteome, a variety of pathways and cellular processes could have been impacted.

### 2.3. Identification of Candidate CAPN5 Substrates with N-Terminomics

We employed the Terminal amine isotopic labeling of substrates (TAILS) N-terminomics method to quantitatively compare the levels of N-terminal peptides in parental and *CAPN5^−/−^* SH-SY5Y cells. When an N-terminal peptide that corresponds to an internal sequence of a nascent protein decreases in *CAPN5^−/−^* SH-SY5Y cells, it identifies a likely proteolytic substrate of CAPN5.

The α-amino groups of the N-terminal amino acid residues of human proteins are often modified, mostly by acetylation [30]. The formaldehyde treatment in our workflow modified the primary amino groups of proteins, including unmodified α-amino groups of the N-terminal amino acid residues and the ε-amino groups of lysine side chains. When trypsin hydrolyzes peptide bonds, it generates novel N-termini with exposed α-amino groups. The tryptic peptides with such free N-terminal primary amines were depleted with an aldehyde-derivatized polymer, leaving only those peptides in solution that represented the N-termini of the lysate proteins (Figure 1B). The removal of non-N-terminal tryptic peptides greatly facilitates the identification of the N-terminal peptides by LC-MS/MS.

**Figure 2:**
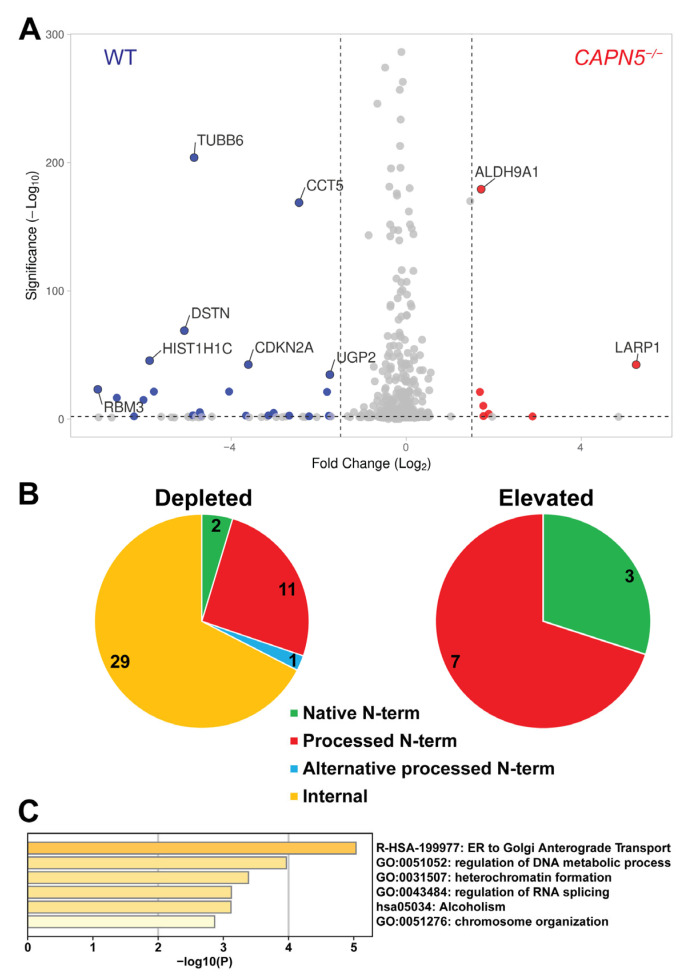
The identification of CAPN5 substrate candidates with TAILS. (**A**) The peptides identified by TAILS are visualized by the volcano plot. Significant outlier cutoff values were determined after log(2) transformation by boxplot-and-whiskers analysis. Significance is defined as −log10(p), shown here with a cutoff > 2. The grey dots represent peptides that were either at similar levels in the parental and *CAPN5*-deficient SH-SY5Y lysates or were detected with a significance under the cutoff. (**B**) N-terminal peptides that were detected by TAILS at significantly lower or higher levels in the *CAPN5^−/−^* SH-SY5Y cells, as compared to the parental SH-SY5Y cells. One of the 30 depleted internal peptides could represent an alternative processed N-terminus. (**C**) Pathway and annotation term analysis of the 29 putative CAPN5 substrates in Table 1 by Metascape.

After the removal of three known contaminant peptides and two peptides that did not match any reviewed human protein entry in the UniProt database, we identified a total of 43 TAILS peptides that were significantly depleted in the *CAPN5^−/−^* SH-SY5Y cells (Table S3, Figure 2A,B). Of these, two were N-terminal peptides including the start methionine residue, and 11 represented N-terminal peptides of the respective proteins with a processed start methionine. Thirty peptides corresponded to internal protein sequences, although one of the peptides may represent a methionine-processed alternative N-terminus of the UGP2/UTP-glucose-1-phosphate uridylyltransferase [31,32] (Figure 2B). It is worth noting that these peptides were rather scarce in the pre-TAILS sample. We could detect only 10 of these 30 peptides in the pre-TAILS shotgun proteomics results, and all 10 were present at such low levels that it precluded their quantification in that dataset.

Twenty-five peptides unambiguously identified unique proteins in the human proteome (Table 1). Histone H4 was the only protein that was identified by two unique internal peptides, corresponding to two separate cleavage sites. Identical Histone H4 proteins are encoded by 14 human genes, of which *H4C12* is expressed highest at the mRNA level in SH-SY5Y cells. Accordingly, *H4C12* was included in further analysis.

Five of the internal peptides could not be unambiguously assigned to unique proteins, but rather to closely related members of protein families (Table S4). One of the peptides may have been derived from any of 15 members of the Histone H2A family [33] that are encoded by a total of 21 genes. Of these, the *H2AZ1* gene encoding Histone H2A.Z is expressed the highest in SH-SY5Y cells at the mRNA level. Another non-unique peptide could have been derived from heat shock proteins HSP90-alpha or HSP90-beta [34], encoded by genes *HSP90AA1* or *HSP90AB1*, respectively, of which the latter is expressed slightly higher at the mRNA level in SH-SY5Y cells. The HSP90 peptide also could have been derived from putative heat shock protein HSP 90-beta-3, although the corresponding gene *HSP90AB3P* is a possible pseudogene. Two members of the Speedy/RINGO protein family, E2B and E6 [35,36], could have both contributed one of the internal peptides, with the mRNA encoding the E2B protein expressed higher in SH-SY5Y cells. Proteolytic processing of three members of the Tubulin beta family, the beta-4A chain, the beta-4B chain, or the beta-8 chain [37], encoded by genes *TUBB4A*, *TUBB4B* and *TUBB8*, respectively, all could have resulted in another non-unique peptide. Of these, the *TUBB4B* mRNA is expressed at the highest level in SH-SY5Y cells. In a separate study, we confirmed the Tubulin beta-4B chain as a CAPN5 interaction partner and in vitro substrate (Gal et al., data from a manuscript in preparation). An internal peptide could have been derived from Trypsin-1, Trypsin-2, or Trypsin-6 [38,39]. Of these, the gene encoding Trypsin-2 is expressed higher in SH-SY5Y cells, and the gene corresponding to Trypsin-6 may be a pseudogene. It should be noted that the identified peptide corresponds to human trypsins. The corresponding peptide of the porcine trypsin that was used to passage the cells and to perform the tryptic digests differs from the identified peptide at a single residue. In Table 1 and in the analysis below, we included the highest expressed genes and the corresponding proteins that could have contributed the respective ambiguous peptides.

Metascape analysis [29] of the genes encoding the putative CAPN5 substrates (Table 1) showed that the terms and pathways “ER to Golgi Anterograde Transport”, “regulation of DNA metabolic process”, “heterochromatin formation”, “regulation of RNA splicing” and “chromosome organization” were significantly enriched (Figure 2C).

We also identified 10 TAILS peptides that were significantly enriched in the *CAPN5^−/−^* SH-SY5Y cells (Table S3). Three of these peptides were N-terminal peptides of the respective proteins including the start methionine residue, and seven started with the second residue of N-terminal peptides of the corresponding proteins, representing processed start methionine residues (Figure 2B). These 10 N-terminal peptides likely represent proteins whose levels were elevated as a response to the loss of CAPN5.

Next, we investigated the interaction of selected substrate candidates with CAPN5 in co-immunoprecipitation, their proteolysis by CAPN5 in an in vitro assay, and compared their fragmentation in parental and *CAPN5^−/−^* SH-SY5Y cells. These confirmatory experiments were performed as single experiments with the necessary controls, as detailed below.

### 2.4. Confirmation of the Interaction Between CAPN5 and Its Potential Substrates

Nine potential CAPN5 substrates identified with N-terminomics were chosen for further evaluation. To confirm the interactions between CAPN5 and its putative substrates, 3×HA-tagged expression constructs of the substrates were generated. Except for 3×HA-MPDZ, the 3×HA tags were present at the C-termini of the proteins. The 3×HA-tagged expression constructs were co-transfected with a wild-type (WT) CAPN5-3×FLAG expression construct [6] into SH-SY5Y cells, followed by immunoprecipitation (IP) with an anti-HA resin in the absence of free Ca^2+^. The bound proteins were eluted by heating the washed resin in the presence of SDS-PAGE loading buffer.

**Figure 3:**
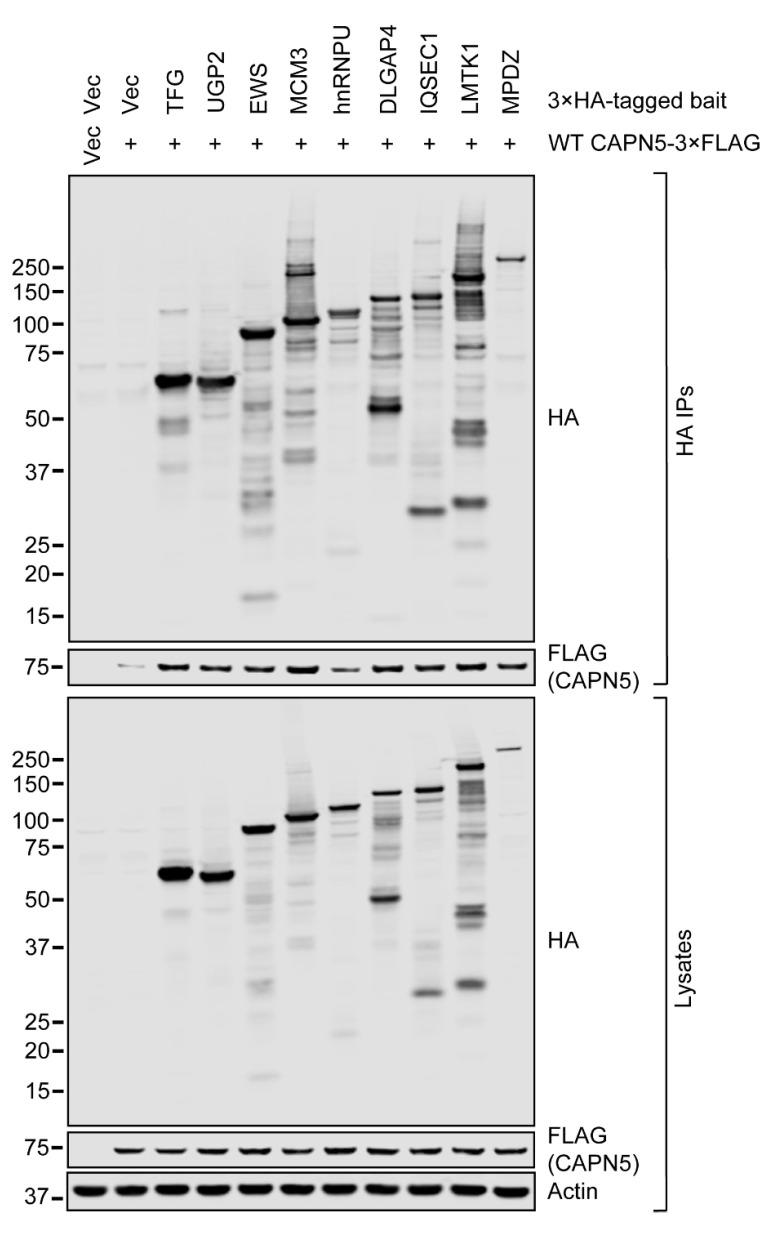
The co-precipitation of WT CAPN5-3×FLAG with its 3×HA-tagged putative substrates in anti-HA immunoprecipitation. SH-SY5Y cells were co-transfected with expression constructs for WT CAPN5-3×FLAG and the indicated 3×HA-tagged proteins or their respective vector controls, as indicated, followed by anti-HA immunoprecipitation, denaturing protein gel electrophoresis and immunoblotting with anti-FLAG antibody to detect CAPN5-3×FLAG, anti-HA antibody to detect the 3×HA-tagged substrate candidates and anti-actin as a loading control. The bars indicate molecular weight marker bands (kDa).

The expression of CAPN5-3×FLAG (“prey”) and the 3×HA-tagged proteins (“bait”) were controlled by their respective empty vectors. The baseline affinity of CAPN5-3×FLAG to the anti-HA immunoaffinity resin was controlled by the 3×HA vector control. Immunoblotting of the lysates showed that the expression of all nine 3×HA-tagged proteins was detectable at the expected molecular weights (Figure 3). HA-immunoreactive bands were also present at lower molecular weights in the lysates of the cells that were transfected with the 3×HA-tagged constructs, likely representing proteolytic products of the respective proteins.

The CAPN5-3×FLAG protein was detected at comparable levels in the lysates when it was co-transfected either with the insert-free 3×HA expression vector or the 3×HA-tagged protein expression constructs. CAPN5-3×FLAG was barely detectable in the immunoprecipitate when it was co-transfected with insert-free 3×HA vector. Compared to the 3×HA vector control, CAPN5-3×FLAG was apparently enriched in all immunoprecipitates when it was co-transfected with the 3×HA-tagged protein expression constructs. The lowest enrichment was seen with the hnRNPU-3×HA bait. Notably, the immunoprecipitated full-length hnRNPU-3×HA band was one of the weakest among the baits. Although the immunoprecipitated 3×HA-MPDZ band was at the lowest intensity among the baits, the enrichment of CAPN5-3×FLAG was comparable to that with the other baits.

Taken together, our anti-HA co-immunoprecipitation results confirmed the interaction between the tested proteins and CAPN5 in vitro.

### 2.5. In Vitro CAPN5 Assays of the Substrate Candidates

Next, the above nine 3×HA-tagged proteins were tested in an in vitro CAPN5 proteolysis assay [11]. The 3×HA-tagged expression constructs were co-transfected with WT or catalytically inactive/dead C81A mutant CAPN5-3×FLAG or insert-free 3×FLAG vector control into SH-SY5Y cells. The expression of the CAPN5-3×FLAG (“bait”) and the 3×HA-tagged (“prey”) proteins were controlled by their respective empty vectors. The baseline affinity of the 3×HA-tagged proteins to the anti-FLAG immunoaffinity resin was controlled by co-transfection with the 3×FLAG vector control. The electrophoretic mobilities of the full-length proteins corresponded to the expectations. Please note that the anti-FLAG immunoprecipitations confirm the same protein–protein interactions as the anti-HA immunoprecipitations in Figure 3, but with reversed prey/bait arrangement. The calcium activation in the in vitro CAPN5 assays were confirmed by CAPN5 autolysis shown by the loss of full-length WT, but not C81A CAPN5, and also by the appearance of C-terminal autolytic WT CAPN5-3×FLAG fragments between approx. 10–17 kDa (please see the high-brightness anti-FLAG immunoblots of the immunoprecipitations, submitted as Supplemental Data).

The C81A mutant served as a positive control for the CAPN5 interaction, but as negative control for CAPN5 proteolytic activity. The lysates were prepared in the presence of 1 mM EDTA to chelate free Ca^2+^ and subjected to immunoprecipitation with anti-FLAG resin. The bound proteins were eluted among non-denaturing (native) conditions with 3×FLAG peptide. Aliquots of the eluates were supplemented with 3 mM CaCl_2_ which, when combined with the 1 mM EDTA in the elution buffer, resulted in approximately 2 mM free Ca^2+^. After a 2 h incubation at room temperature, the eluates were analyzed with denaturing gel electrophoresis and immunoblotting.

**Figure 4:**
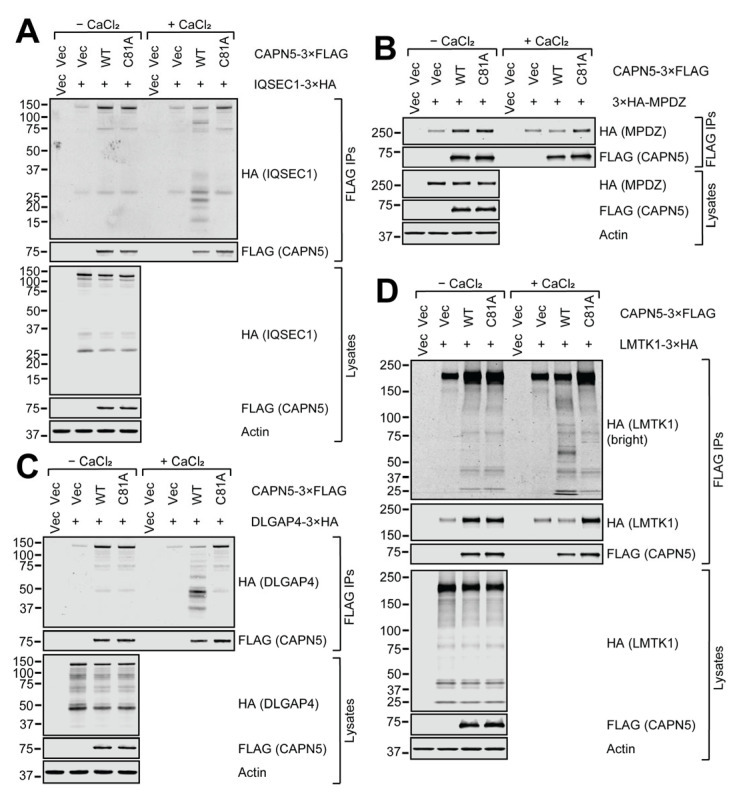
In vitro CAPN5 assay of putative CAPN5 substrates IQSEC1 (**A**), MPDZ (**B**), DLGAP4 (**C**) and LMTK1 (**D**). SH-SY5Y cells were co-transfected with expression constructs for WT or C81A mutant CAPN5-3×FLAG and 3×HA-tagged putative substrate proteins or their respective vector controls. The cellular lysates were subjected to anti-FLAG immunoprecipitation, and the bound proteins were eluted among native conditions with 3×FLAG peptide. Aliquots of the eluates were incubated with or without activating CAPN5 by the addition of CaCl_2_ followed by denaturing protein gel electrophoresis and immunoblotting with anti-FLAG antibody to detect CAPN5-3×FLAG, anti-HA antibody to detect the respective 3×HA-tagged substrate candidates (indicated in parentheses) and anti-actin as a loading control. The bars indicate molecular weight marker bands (kDa).

The 3×HA-tagged IQSEC1, DLGAP4, MPDZ and LMTK1 proteins were all clearly enriched by the WT and C81A CAPN5-3×FLAG baits compared to the 3×FLAG vector control (Figure 4). Upon calcium addition, the bands that correspond to the full-length co-precipitated 3×HA-tagged proteins got weaker in the presence of WT CAPN5. For IQSEC1, DLGAP4 and LMTK1, lower molecular weight (MW) proteolytic fragments were also apparent. The 3×HA-MPDZ protein was the only example among the nine proteins tested where no proteolytic fragmentation was detected with WT CAPN5-3×FLAG. It suggests that CAPN5 may have had at least one proteolytic cut site close to the N-terminus of MPDZ that resulted in fragments that were too short to be detected. It is also possible that CAPN5 could have had several proteolytic cut sites within MPDZ, diluting the signal enough to make the fragments undetectable.

**Figure 5:**
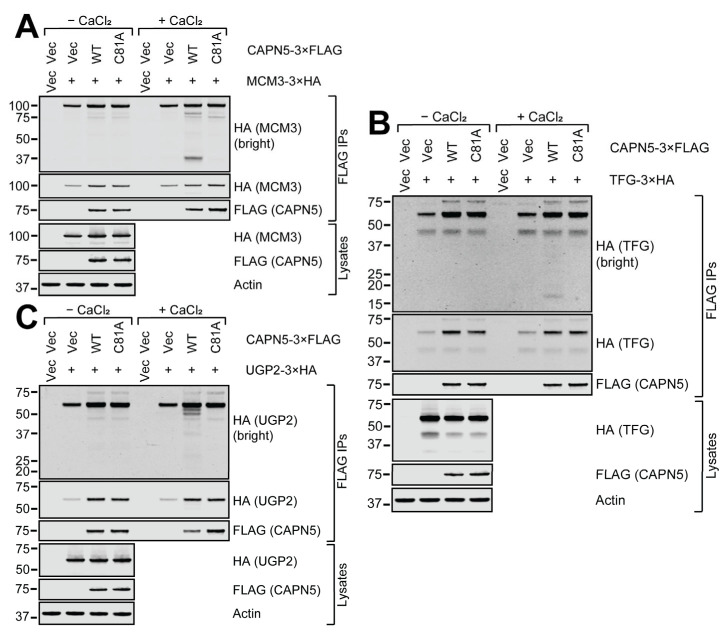
In vitro CAPN5 assay of putative CAPN5 substrates MCM3 (**A**), TFG (**B**) and UGP2 (**C**). Please see the main text and the legend of Figure 4 for details. The bars indicate molecular weight marker bands (kDa).

The TFG-3×HA and the UGP2-3×HA proteins were significantly enriched by the CAPN5-3×FLAG baits, whereas the enrichment of MCM3-3×HA was more modest (Figure 5). Although the loss of the full-length 3×HA-tagged bands upon calcium addition was less prominent, low MW proteolytic fragments of all three proteins were detectable.

**Figure 6:**
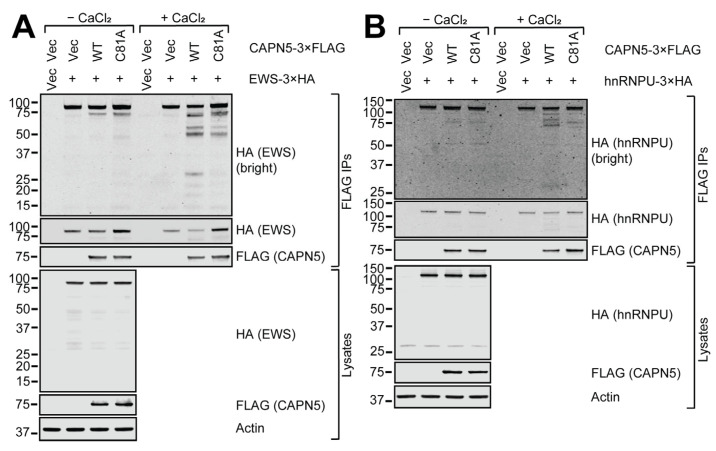
In vitro CAPN5 assay of putative CAPN5 substrates EWS (**A**) and hnRNPU (**B**). Please see the main text and the legend of Figure 4 for details. The bars indicate molecular weight marker bands (kDa).

The 3×HA-tagged EWS protein showed significant affinity to the anti-FLAG resin (Figure 6A). The enrichment of the EWS-3×HA protein was only apparent with the C81A mutant CAPN5-3×FLAG bait. Upon calcium addition, both the loss of full-length EWS-3×HA and the appearance of smaller proteolytic fragments were clear with WT CAPN5-3×FLAG. Unexpectedly, proteolytic fragments around 50 kDa were also detected in the presence of C81A CAPN5-3×FLAG. These were likely due to a co-precipitating protease activity, since the C81A CAPN5 mutant is proteolytically dead [6].

The hnRNPU protein also showed significant affinity to the anti-FLAG resin, and it was not appreciably enriched by the CAPN5-3×FLAG baits. However, calcium induction did result in a modest loss of full-length hnRNPU-3×HA and the appearance of low MW proteolytic products with WT CAPN5-3×FLAG (Figure 6B).

### 2.6. Cellular Fragmentation of the Putative CAPN5 Substrates

**Figure 7:**
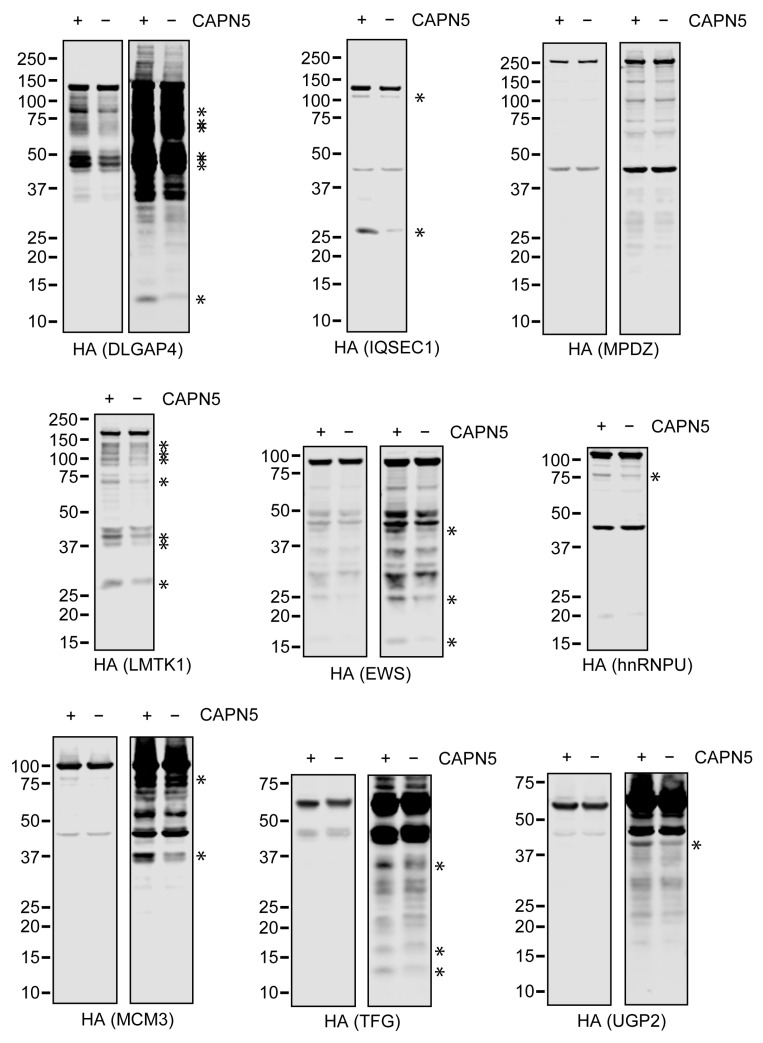
The cellular fragmentation of the 3×HA-tagged putative CAPN5 substrates. Parental or *CAPN5^−/−^* SH-SY5Y cells were transfected with expression constructs for the respective 3×HA-tagged putative CAPN5 substrates, followed by lysate preparation, denaturing protein gel electrophoresis and immunoblotting with anti-HA antibody to detect the respective 3×HA-tagged CAPN5 substrate candidates (indicated in parentheses). The loading was equalized for equally intense full-length proteins in the parental and the *CAPN5^−/−^* cells. For some of the proteins, a pair of images are shown at lower and higher brightness. Prominent differences between the fragment intensities in the parental and *CAPN5^−/−^* SH-SY5Y cells are highlighted with asterisks. The bars indicate molecular weight marker bands (kDa).

To determine the effect of the CAPN5 status on the proteolytic fragmentation of the tested proteins in living cells, parental and *CAPN5^−/−^* SH-SY5Y cells were transfected with the 3×HA-tagged expression constructs, followed by lysate preparation and immunoblotting (Figure S1). The expression of the 3×HA-tagged proteins were controlled by the transfection of empty 3×HA-tagging expression vector. To facilitate the comparison of the fragmentation patterns, the loading was repeated with normalized full-length protein levels (Figure 7).

Immunoblotting of the lysates with anti-HA antibody showed that the fragmentation of some of the tested proteins was apparently stronger in the parental SH-SY5Y cells than in the *CAPN5^−/−^* cells (Figure 7). Some of the protein bands that were more intense in the parental SH-SY5Y cellular lysates were similar to proteolytic fragments that were detected in the in vitro CAPN5 assays. These included DLGAP4 (under 50 kDa), IQSEC1 (above 25 kDa), LMTK1 (between 100 and 150 kDa), hnRNPU (above 75 kDa), MCM3 (at 37 kDa and above 75 kDa), TFG (above 15 kDa) and EWS (at 25 kDa).

## 3. Discussion

The substrate repertoire of CAPN5 remains largely uncharacterized. The identification of calpain substrates has proven difficult, complicated by the lack of clear proteolytic consensus sequences. The TAILS N-terminomics method is an unbiased and quantitative tool to identify protease substrates as demonstrated previously [4,40,41,42,43]. This study identified 24 unique proteins and closely related members of five protein families as candidate substrates of CAPN5 by comparing the N-terminomes of parental and *CAPN5^−/−^* SH-SY5Y neuroblastoma cells. Follow-up studies provided evidence for the proteolysis of a subset of the candidate substrates: we confirmed their interaction with CAPN5, in vitro proteolysis by CAPN5, and decreased fragmentation in *CAPN5^−/−^* SH-SY5Y cells.

Calpains are regulatory proteases that carry out proteolysis only of a limited subset of proteins. Our pre-TAILS shotgun proteomics results suggested that the inactivation of *CAPN5* altered the levels of only a small subset of the total proteome, as expected. The altered protein levels may either be caused by CAPN5-mediated proteolysis, or they may reflect physiological adaptations of the *CAPN5^−/−^* cells. Nevertheless, it is worth noting that we detected the SLIT2 protein in pre-TAILS proteomics only with a peptide that was significantly enriched in the *CAPN5^−/−^* cells, and SLIT2 is one of the few previously reported substrates of CAPN5 [20].

An advantage of the TAILS N-terminomics method is that it is not based on the affinity of a protease to its substrate. Substrates whose affinity to the protease is relatively low may be difficult to detect with methods such as co-precipitation. Affinity-based methods can also detect interaction partners of proteases that are not substrates of the protease. However, N-terminomics also has its limitations. Proteolysis of a substrate may result in destabilization and rapid turnover of the proteolytic products in living cells, precluding their detection by N-terminomics. Therefore, only stable cleaved proteins are likely to be identified via N-terminomics. Another limitation of the TAILS method is that it cannot determine the total size of the detected proteolytic fragments, only the positions of the neo-N-termini. Our in vitro CAPN5 assays detected multiple proteolytic fragments with most tested CAPN5 substrate candidates, suggesting that there were multiple cleavage sites per protein. It may explain discrepancies between predicted and detected proteolytic fragment sizes. Some of the small proteolytic fragments may also have been difficult to detect with immunoblotting.

Our primary goal in this study was to identify proteolytic substrates of CAPN5 in the central nervous system. We conducted our studies in the SH-SY5Y human neuroblastoma cell line [22]. However, it is also another limitation of our study that the substrate repertoire of CAPN5 may be significantly different in other cell types and among other experimental conditions.

TAILS identified 43 peptides that were depleted in the *CAPN5^−/−^* cells, the majority of which represented internal peptides of nascent proteins. Conversely, none of the 10 TAILS peptides that were identified as elevated in *CAPN5^−/−^* cells were internal. It suggests that the internal TAILS peptides that were depleted in the *CAPN5^−/−^* cells were likely produced by CAPN5 proteolysis. One of the depleted internal peptides that correspond to UGP2 might represent a processed alternative N-terminus of the protein [31,32]. However, the result of the in vitro CAPN5 assay of full-length UGP2-3×HA (Figure 5C) was consistent with proteolytic trimming very close to the N-terminus of the protein. It suggests that multiple mechanisms could result in the “short” form of UGP2 at the protein level.

The fragmentation patterns of the tested CAPN5 candidates showed differences between the in vitro CAPN5 assays (Figure 4, Figure 5 and Figure 6) and in living cells (Figure 7). After initial proteolytic cleavage(s) by CAPN5, the generated proteolytic fragments may undergo further proteolysis in living cells. The C-terminal fragments resulting from substrate proteolysis by classical calpains were predicted to be short-lived due to their being targeted for the ubiquitin-proteasome system as a result of the Arg/N-end rule [44].

Many CAPN5 substrate candidates identified in this study harbor genetic variants that cause human diseases including several neurodevelopmental disorders [32,45,46,47,48,49,50,51,52,53,54,55,56,57,58,59,60,61,62,63,64,65,66,67,68,69,70,71] (Table 2). It suggests that abnormally high or low CAPN5 activity may contribute to pathogenesis. The dysregulation of calpains, primarily their hyperactivation, was reported in a variety of human diseases [72,73,74].

Neovascular inflammatory vitreoretinopathy (NIV) is a rare, progressive, inflammatory autoimmune eye disease that leads to blindness [75,76]. NIV is caused by mutations in *CAPN5* [12,14,20,77,78]. NIV has characteristics that mimic several other eye diseases including autoimmune eye diseases and retinal neurodegeneration [12]. We reported that the autoimmune regulator AIRE specifically interacted with CAPN5, and it was also an in vitro CAPN5 substrate that could be relevant to the autoimmune component of the disease [11]. This study identified several additional CAPN5 targets with potential relevance to retinal neurodegeneration and neurologic disorders.

CAPN5 was detected in photoreceptor synapses in the retina, and the electroretinogram of NIV patients showed defective retinal synaptic signaling [8]. Three CAPN5 substrate candidates identified in this study, IQSEC1, MPDZ and DLGAP4, play important roles in synaptic processes.

IQSEC1 is a core component of the postsynaptic density at excitatory synapses in the brain [47]. IQSEC1 is involved in neuronal development and mediates the activation of ARF6 to internalize synaptic AMPARs [47]. The *Drosophila* ortholog of IQSEC1 is schizo whose loss affects growth cone guidance at the midline in the CNS, and it is required in photoreceptors for phototransduction [47]. In the mouse retina, IQSEC1 is localized to photoreceptor terminals in rod spherules and cone pedicles. In the inner plexiform layer, IQSEC1 localized to synapses and colocalized with PSD95/DLG4 and glutamate receptors (AMPARs) [79]. MPDZ is a member of the NMDAR signaling complex that may play a role in control of AMPAR potentiation and synaptic plasticity in excitatory synapses [80,81]. It is a peripheral membrane protein localized to the cell membrane, highly enriched at postsynaptic densities. It colocalizes with the synaptic marker PSD95. In the retina, MPDZ localizes to the sub-apical region adjacent to the adherens junction complex at the outer limiting membrane. Its mutations cause hydrocephalus, congenital 2, a disease that can present with eye anomalies [45,67]. An MPDZ mutation was identified as the cause of an inherited retinal dysplasia and degeneration in chicken, and MPDZ variants were identified in human patients with retinitis pigmentosa and Leber congenital amaurosis that are diseases of the eye [46]. DLGAP4 plays important roles at synapses as a component of the PSD95–SAPAP–SHANK excitatory postsynaptic scaffolding complex [82].

Although our N-terminomics screen could not determine whether an internal peptide was derived from TUBB4A, TUBB4B or TUBB8 (Table S4), we recently confirmed the specific interaction between TUBB4B and CAPN5, and vitro proteolysis of TUBB4B by CAPN5 (Gal et al., manuscript in preparation). The Tubulin beta-6 chain/TUBB6 was identified with a unique internal peptide by TAILS (Table 1). The closest homolog of CAPN5 is CAPN6, the only proteolytically inactive human calpain [5]. Interestingly, CAPN6 was found to be a microtubule-stabilizing protein [83].

*TUBB4B* was reported to harbor variants that cause Leber congenital amaurosis, an eye disease, with early-onset deafness [63]. A variant in a gene that encodes another CAPN5 substrate candidate, *SPTBN4*, was reported to cause neurodevelopmental disorder with hypotonia, neuropathy and deafness [62]. Hearing loss was also reported in NIV [14], suggesting that it could be caused by dysregulated proteolysis of TUBB4B and/or SPTBN4 by CAPN5.

## 4. Materials and Methods

### 4.1. Cell Culture and the Generation of the CAPN5^−/−^ SH-SY5Y Cell Line

The SH-SY5Y human neuroblastoma cell line (ATCC, Manassas, VA, USA, CRL-2266 [22]) was maintained in MEM with Earle’s salts and L-glutamine (Corning, Corning, NY, USA, 10-010-CV) supplemented with 10% fetal bovine serum (FBS) (Atlanta Biologicals, Flowery Branch, GA, USA, S11195H) and penicillin–streptomycin (Corning, 30-002-CI). The cells were maintained in humidified cell culture incubator under 5% CO_2_/95% air at 37 °C.

The SH-SY5Y cells were transfected with Calpain-5 Double Nickase CRISPR Plasmid (Santa Cruz Biotechnology, Dallas, TX, USA, sc-403881-NIC) using the LipoJet DNA In Vitro Transfection Reagent (Signagen, Frederick, MD, USA, SL100468). The transfected cells were selected with 5 μg/mL puromycin dihydrochloride (Gold Biotechnology, St. Louis, MO, USA, P-600-100) in regular medium for five days to remove the untransfected cells. The cells were allowed to grow to confluence in regular medium, followed by single-cell cloning by plating the cells into wells of a 96-well plate at a density of 0.5 live cells per well. Until colonies formed, the medium was modified to contain 30% FBS and 25% sterile-filtered conditioned SH-SY5Y medium. The colonies were expanded, and cellular lysates were prepared in 1× RIPA buffer (50 mM Tris-HCl, pH 7.4, 150 mM NaCl, 0.25% deoxycholic acid, 1% NP-40 and 1 mM EDTA; Sigma, St. Louis, MO, USA, 20–188) supplemented with protease inhibitor cocktail (Sigma, P8340, 1:300) and 1 mM sodium orthovanadate. The CAPN5 status of individual clones was determined with immunoblotting, and a cell line with no detectable CAPN5 expression was chosen for further studies (Figure 1A).

### 4.2. Sample Preparation for Proteomics

Parental and *CAPN5^−/−^* SH-SY5Y cells were cultured to confluence on 10 cm tissue culture dishes. The dishes were rinsed three times with 1× PBS (137 mM NaCl, 2.7 mM KCl, 10 mM Na_2_HPO_4_, 1.8 mM KH_2_PO_4_, pH 7.4), and the cells were directly suspended into lysis buffer (100 mM NH_4_HCO_3_, 1% sodium dodecyl sulfate, 0.2 mM EDTA) supplemented with protease inhibitor cocktail (1:500, Sigma, P8340). The viscosity of the samples was reduced by passing them through 18-gauge, then 23-gauge needles several times, followed by sonication. The lysates were centrifuged at 14,000 × g for 10 min at 4 °C. The supernatants were transferred to fresh tubes and the protein concentrations were measured using a NanoPhotometer (Implen, N50).

### 4.3. N-Terminomics/Terminal Amine Isotopic Labeling of Substrates (TAILS) and Shotgun Proteomics

The lysates were subjected to an N-terminomics/TAILS workflow [41,43,84,85,86,87,88,89,90]. Briefly, the protein concentrations were normalized with lysis buffer, and the samples were reduced with 5 mM DTT at 37 °C for 1 h, then alkylated with 15 mM iodoacetamide in the dark at room temperature for 30 min, followed by quenching with 15 mM DTT. The pH was adjusted to 6.5 with HCl before the samples were isotopically labeled with a final concentration of 40 mM light formaldehyde (parental SH-SY5Y) or 40 mM deuterated heavy formaldehyde (*CAPN5^−/−^* SH-SY5Y) in the presence of 40 mM sodium cyanoborohydride at 37 °C overnight. Next, the samples were combined and were precipitated using acetone/methanol (8:1 [*v*:*v*]). The resulting pellet was resuspended, the pH was adjusted to 8.0 with NaOH and the proteins were subjected to trypsin digestion at 37 °C overnight. For pre-enrichment TAILS (pre-TAILS)/shotgun proteomics, 10% of the trypsin-digested samples were adjusted to pH 3.0 with formic acid. The rest of the samples were adjusted to pH 6.5 and incubated with a 3-fold excess [*w*/*w*] of dendritic polyglycerol aldehyde polymer at 37 °C overnight [43,86,91]. Unbound peptides were filtered out with ultrafiltration through 10 kDa cutoff membrane (Amicon Ultra, Millipore, Burlington, MA, USA) with centrifugation at 10,000× *g* for 5 min. The flow-through was collected and the ultrafiltration columns were washed with 100 mM Tris-HCl, pH 6.5. The samples were adjusted to pH 3.0 with formic acid. Both pre-TAILS and TAILS samples were then desalted using Sep-Pak C18 columns and lyophilized before submitting for LC-MS/MS analysis to the Southern Alberta Mass Spectrometry Core Facility, University of Calgary, Canada.

### 4.4. High Performance Liquid Chromatography (HPLC) and Mass Spectrometry

Analysis was performed on an Orbitrap Fusion Lumos Tribrid mass spectrometer (Thermo Fisher Scientific, Waltham, MA, USA) operated with Xcalibur (version 4.0.21.10) and coupled to a Thermo Fisher Scientific Easy-nLC (nanoflow Liquid Chromatography) 1200 system. Tryptic peptides (2 µg) were loaded onto a C18 trap (75 µm × 2 cm; Acclaim PepMap 100, Thermo Fisher Scientific, 164946) at a flow rate of 2 µL/min of solvent A (0.1% formic acid and 3% acetonitrile in LC-mass spectrometry grade water). Peptides were eluted using a 120 min gradient from 5 to 40% (5% to 28% in 105 min, followed by an increase to 40% B in 15 min) of solvent B (0.1% formic acid in 80% LC–mass spectrometry grade acetonitrile) at a flow rate of 0.3 µL/minute and separated on a C18 analytical column (75 µm × 50 cm; PepMap RSLC C18; Thermo Fisher Scientific, ES803). Peptides were then electrosprayed using 2.3 kV into the ion transfer tube (300 °C) of the Orbitrap Lumos operating in positive mode. The Orbitrap first performed a full mass spectrometry scan at a resolution of 120,000 FWHM to detect the precursor ion having a mass-to-charge ratio (*m*/*z*) between 375 and 1575 and a +2 to +4 charge. The Orbitrap AGC (Auto Gain Control) and the maximum injection time were set at 4 × 10^5^ and 50 ms, respectively. The Orbitrap was operated using the top speed mode with a 3 s cycle time for precursor selection. The most intense precursor ions presenting a peptidic isotopic profile and having an intensity threshold of at least 2 × 10^4^ were isolated using the quadrupole (isolation window of *m*/*z* 0.7) and fragmented with HCD (38% collision energy) in the ion routing Multipole. The fragment ions (MS2) were analyzed in the Orbitrap at a resolution of 15,000. The AGC, the maximum injection time and the first mass were set at 1 × 10^5^, 105 ms and 100 ms, respectively. Dynamic exclusion was enabled for 45 s to avoid the acquisition of the same precursor ion having a similar *m*/*z* (±10 ppm).

### 4.5. Proteomic Data and Bioinformatic Analysis

Spectral data were matched to peptide sequences in the human UniProt protein database using the MaxQuant software package v.1.6.23, peptide-spectrum match false discovery rate (FDR) of <0.01 for the shotgun proteomics data and <0.05 for the N-terminomics/TAILS data. Search parameters included a mass tolerance of 20 ppm for the parent ion, 0.05 Da for the fragment ion, carbamidomethylation of cysteine residues (+57.021464), variable N-terminal modification by acetylation (+42.010565 Da) and variable methionine oxidation (+15.994915 Da). For the N-terminomics/TAILS data, the cleavage site specificity was set to semi-ArgC (search for free N-terminus) for the TAILS data and was set to ArgC for the preTAILS data, with up to two missed cleavages allowed. Significant outlier cutoff values were determined after log(2) transformation by boxplot-and-whiskers analysis using the BoxPlotR tool [28]. Database searches were limited to a maximal length of 40 residues per peptide. Peptide sequences matching reverse or contaminant entries were removed. The mass spectrometry proteomics data have been deposited to the ProteomeXchange Consortium [92] via the PRIDE partner repository [93] with the dataset identifier PXD064313.

### 4.6. Plasmid Construction

The construction of the plasmids expressing WT or C81A mutant CAPN5-3×FLAG, based on p3×FLAG-CMV-14 (Sigma, E4901) was reported before [6]. The 3×HA-tagged expression constructs of the CAPN5 substrate candidates were made by Gateway recombination using the LR Clonase II Enzyme Mix (Thermo Fisher Scientific, 11791020). The entry clone to generate the 3×HA-MPDZ expression construct was obtained from Horizon Discovery (Cambridge, UK, clone OHS5893-202503824), and the destination vector was pGCS-N2(3×HA) (a gift from Hai-Ning Du, Addgene (Watertown, MA, USA) plasmid # 85719, http://n2t.net/addgene:85719 (accessed on 23 August 2021); RRID:Addgene_85719 [94]). The destination vector to generate all other 3×HA-tagged expression constructs was pCSF107mT-GATEWAY-3’-3HA, which confers a C-terminal 3×HA-tag (a gift from Todd Stukenberg (Addgene plasmid # 67616, http://n2t.net/addgene:67616 (accessed on 23 August 2021); RRID:Addgene_67616). The entry clone to generate EWS-3×HA was pDONR221-EWSR1 No Stop (a gift from Aaron Gitler, Addgene plasmid # 84888, http://n2t.net/addgene:84888 (accessed on 14 September 2021); RRID:Addgene_84888 [95]). The entry clone for the hnRNPU-3×HA expression construct was from Horizon Discovery (clone OHS5894-202498112). All other entry clones were obtained from DNASU (Tempe, AZ, USA) [96,97,98], and are summarized in Table 3. The Hemagglutinin (HA) tag sequence (YPYDVPDYA) was identical in the 3×HA-tagging expression vectors, with differences in the spacing between the tags. In pGCS-N2(3×HA), the three HA-tags are in tandem with no linker between them. In pCSF107mT-GATEWAY-3’-3HA, the first and second HA-tags are separated by a glycine residue, and the second and third HA-tags are separated by a glycine–serine linker. The recombination junctions were confirmed with DNA sequencing.

### 4.7. Anti-HA Immunoprecipitation

Parental SY-SY5Y cells were co-transfected with expression constructs for WT CAPN5-3×FLAG and the 3×HA-tagged substrate candidate proteins or the respective vector controls using the LipoJet DNA In Vitro Transfection Reagent. Two days later, the transfected cells were rinsed with 1× PBS, and cellular lysates were prepared in ice-cold 1× RIPA buffer supplemented with protease inhibitor cocktail (Sigma, P8340, 1:300) and phosphatase inhibitor cocktail (PhosSTOP, Sigma, 4906837001, 1 tablet per 10 mL). The downstream steps were performed on ice, unless otherwise indicated. The lysates were passed through 23-gauge needles several times, incubated for 20 min with short vortexing at 10 and 20 min, and cleared with centrifugation at 8200× *g* for 10 min at 4 °C. The supernatants were transferred to fresh tubes, and the protein concentrations of the samples were determined with Protein Assay Dye Reagent (Bio-Rad, Hercules, CA, USA, 500-0006). The protein concentrations were normalized with lysis buffer, followed by immunoprecipitation with Anti-HA Magnetic Beads (Sigma, SAE0197, previously washed with lysis buffer) with end-over-end rotation at 4 °C for three hours. Afterwards, the beads were washed with lysis buffer three times. Bound proteins were eluted with 2× Laemmli Sample Buffer (Bio-Rad, 1610737) without reducing agent in a dry block at 60 °C for 10 min, and the eluates were transferred to fresh tubes. Aliquots of the total lysates were also saved with the addition of 6× SDS-PAGE loading buffer (0.35 M Tris-HCl, pH 6.8, 30% [*v*/*v*] glycerol, 12% [*w*/*v*] sodium dodecyl sulfate, 0.6 M dithiothreitol and 0.06% [*w*/*v*] bromophenol blue), and heated at 60 °C for 10 min.

### 4.8. In Vitro CAPN5 Assay

The in vitro CAPN5 assays [11] were performed as follows. The expression constructs for WT or C81A CAPN5-3×FLAG and the 3×HA-tagged substrate candidates or their respective vector controls were co-transfected into SH-SY5Y cells using the LipoJet DNA In Vitro Transfection Reagent. Two days later, the transfected cells were rinsed with 1× PBS, and cellular lysates were prepared in ice-cold 1× RIPA buffer supplemented with 1 mM AEBSF, an irreversible serine protease inhibitor (Gold Biotechnology, A-540-500) and 1 tablet per 10 mL PhosSTOP phosphatase inhibitor cocktail. The downstream steps were performed on ice, unless otherwise indicated. The lysates were passed through 23-gauge needles several times, incubated for 20 min with short vortexing at 10 and 20 min and cleared with centrifugation at 8200 × g for 10 min at 4 °C. The supernatants were transferred to fresh tubes. The protein concentrations of the samples were determined with the Bio-Rad Protein Assay Dye Reagent and normalized with lysis buffer. Immunoprecipitation was performed with Anti-FLAG M2 Magnetic Beads (Sigma, M8823, previously washed with lysis buffer) at 4 °C with end-over-end rotation for three hours. The beads were washed with Calpain Reaction Buffer (50 mM Tris-HCl, pH 7.5, 50 mM NaCl, 1 mM EDTA and 1 mM AEBSF) three times. Bound proteins were eluted among native conditions with 0.1 mg/mL 3×FLAG peptide (Sigma, F4799) in Calpain Reaction Buffer with end-over-end rotation at 4 °C for one hour. Two equal aliquots of each eluate were transferred to low protein binding tubes. CAPN5 was activated by supplementing one aliquot with 3 mM calcium chloride and 5 mM beta-mercaptoethanol (final concentrations), followed by 2 h of incubation at room temperature. The control reactions were supplemented with 5 mM beta-mercaptoethanol only. The reactions were stopped with the addition of 6× SDS-PAGE loading buffer and heated at 60 °C for 10 min. Aliquots of the total lysates were also saved with the addition of 6× SDS-PAGE loading buffer and heating as above.

### 4.9. Denaturing Gel Electrophoresis and Immunoblotting

Equal volumes of the immunoprecipitates and equal protein amounts of the lysates were resolved on 3–8% Tris-acetate gradient protein gels (Thermo Fisher Scientific, EA0378BOX) using Tris-acetate-SDS running buffer, pH 8.24 (Thermo Fisher Scientific, LA0041), or 4–12% Bis-Tris gradient protein gels (Thermo Fisher Scientific, NP0335BOX or NP0336BOX) using MES SDS running buffer, pH 7.3 (Thermo Fisher Scientific, NP0002). The resolved proteins were transferred to nitrocellulose membranes with 0.2 µm pore size (Bio-Rad, 1704158), followed by blocking with blocking buffer (5% non-fat dry milk, 50 mM Tris-HCl, pH 7.5, 0.85% [*w*/*v*] NaCl, 0.1% [*v*/*v*] Tween-20). The primary and secondary antibodies were applied in blocking buffer. The primary antibodies were mouse anti-CAPN5 (Santa Cruz Biotechnology, sc-271271, 1.0 µg/mL), rabbit anti-HA tag (Proteintech, Rosemont, IL, USA, 51064-2-AP, 1.6 µg/mL), rabbit anti-DYKDDDDK tag (FLAG tag, Proteintech, 20543-1-AP, 0.48 µg/mL), mouse anti-FLAG (Sigma, F3165, 2.0 µg/mL) and mouse anti-beta actin (Proteintech, 66009-1-Ig, 0.5 µg/mL). The secondary antibodies were IRDye 800CW goat anti-rabbit IgG (Li-Cor, Lincoln, NE, USA, 926-32211, 0.1 µg/mL), Alexa Fluor 680 goat anti-mouse IgG (Thermo Fisher Scientific, A-21058, 0.2 µg/mL) and IRDye 800CW goat anti-mouse IgG (Li-Cor, 926-32210, 0.1 µg/mL). The images were acquired on a Li-Cor Odyssey CLx Imaging System using the Li-Cor Image Studio software (Version 5.2). The composite immunoblotting images were assembled with Adobe Photoshop 2023 (Version 24.3.0) and Adobe Illustrator 2023 (Version 27.4.1) software. Adjustments to contrast and brightness were applied equally to entire images. Blot lanes were not spliced from different images of a membrane.

### 4.10. Databases

Data about the proteins were gathered from the UniProt database (uniprot.org, release 2022_05, 2022-12-14 [99]). We used the Human Protein Atlas database [100] to gather human cell- and tissue-specific expression information (v23.proteinatlas.org, version: 23.0, updated: 2023-06-19).

## 5. Conclusions

By comparing the proteomes of parental and *CAPN5^−/−^* SH-SY5Y cells, we determined that the levels of only a relatively small subset of proteins were different. By employing the TAILS N-terminomics method, we identified 29 proteolytic substrate candidates of CAPN5 and further characterized nine of them with CAPN5 co-precipitation, in vitro CAPN5 proteolysis assay, and compared their cellular fragmentation in parental and *CAPN5^−/−^* SH-SY5Y cells.

Numerous CAPN5 substrate candidates identified in this study have been implicated in human diseases, suggesting that CAPN5 dysfunction may be involved in pathogenic processes. It will be very important to determine the effect of CAPN5 dysfunction in clinical samples. It is difficult to predict the functional effect of CAPN5 proteolysis of a substrate protein, which is expected to be limited based on substrate proteolysis of other calpains. It may result in activation or inactivation of the substrate, stabilization or destabilization of proteolytic fragments or altered cellular localization. Further studies are necessary to determine the effect of CAPN5 proteolysis of the substrates that were identified in this study.

## Figures and Tables

**Table 1:** Potential CAPN5 substrates identified by TAILS. The proteins were manually assigned to functional groups. *: The respective protein was not unambiguously identified by a TAILS peptide (see the main text and Table S4 for details).

UniProt AC	Gene Name	Protein Name	Cleavage Site
RNA binding proteins			
Q13838	*DDX39B*	Spliceosome RNA helicase DDX39B	355R V356
Q01844	*EWSR1*	RNA-binding protein EWS	614R R615
Q00839	*HNRNPU*	Heterogeneous nuclear ribonucleoprotein U	733R G734
Q9BPZ3	*PAIP2*	Polyadenylate-binding protein-interacting protein 2	47I E48
P98179	*RBM3*	RNA-binding protein 3	101R S102
Synaptic proteins			
Q9Y2H0	*DLGAP4*	Disks large-associated protein 4	944K A945
Q6DN90	*IQSEC1*	IQ motif and SEC7 domain-containing protein 1	158R S159
O75970	*MPDZ*	Multiple PDZ domain protein	1138L Q1139
Cytoskeletal proteins			
Q9H254	*SPTBN4*	Spectrin beta chain, non-erythrocytic 4	855R V856
P68371	*TUBB4B*	Tubulin beta-4B chain *	62R A63
Q9BUF5	*TUBB6*	Tubulin beta-6 chain	62R A63
Regulators of chromosomal processes			
Q8TB52	*FBXO30*	F-box only protein 30	588G V589
P0C0S5	*H2AZ1*	Histone H2A.Z *	23R A24
P62805	*H4C12* (+13)	Histone H4	46R I47, 96R T97
Q6ZMQ8	*AATK*	Serine/threonine-protein kinase LMTK1	403A T404
P25205	*MCM3*	DNA replication licensing factor MCM3	680K S681
O75528	*TADA3*	Transcriptional adapter 3	385R M386
Q9BXT5	*TEX15*	Testis-expressed protein 15	1653R K1654
Q7Z2Z1	*TICRR*	Treslin	186K Q187
Q9H091	*ZMYND15*	Zinc finger MYND domain-containing protein 15	114E G115
Protein processing			
Q9H3G5	*CPVL*	Probable serine carboxypeptidase CPVL	457R A458
P07478	*PRSS2*	Trypsin-2 *	72R L73
Miscellaneous functions			
P27482	*CALML3*	Calmodulin-like protein 3	112R L113
P08238	*HSP90AB1*	Heat shock protein HSP 90-beta *	378R G379
Q86WN2	*IFNE*	Interferon epsilon	160Y S161
O15018	*PDZD2*	PDZ domain-containing protein 2	2574S V2575
A6NHP3	*SPDYE2B*	Speedy protein E2B *	110R V111
Q92734	*TFG*	Protein TFG	383R N384
Q16851	*UGP2*	UTP-glucose-1-phosphate uridylyltransferase	12M S13

**Table 2:** Genes of CAPN5 substrate candidates with variants or genetic rearrangements that were associated with human diseases.

Gene	Disease	Abbreviation, MIM Code	References
*MPDZ*	Hydrocephalus, congenital, 2, with or without brain or eye anomalies	HYC2, MIM:615219	[45,67]
	Leber congenital amaurosis, Retinitis pigmentosa	LCA, RP	[46]
*H4*	Tessadori-van Haaften neurodevelopmental syndrome 1, 2, 3 and 4	TEVANED1–4 MIM:619758, 619759, 619950 and 619951	[68,69,70]
*IQSEC1*	Intellectual developmental disorder with short stature and behavioral abnormalities	IDDSSBA, MIM:618687	[47]
*SPTBN4*	Neurodevelopmental disorder with hypotonia, neuropathy and deafness	NEDHND, MIM:617519	[62]
*HNRNPU*	Developmental and epileptic encephalopathy 54	DEE54, MIM:617391	[52,57]
*UGP2*	Developmental and epileptic encephalopathy 83	DEE83, MIM:618744	[32]
*TFG*	Neuropathy, hereditary motor and sensory, Okinawa type	HMSNO, MIM:604484	[59,61]
	Spastic paraplegia 57, autosomal recessive	SPG57, MIM:615658	[50,58,61,64]
*TUBB4B*	Leber congenital amaurosis with early-onset deafness	LCAEOD, MIM:617879	[63]
*TUBB6*	Facial palsy, congenital, with ptosis and velopharyngeal dysfunction	FPVEPD, MIM:617732	[55]
*EWS*	Ewing sarcoma	ES, MIM:612219	[51,54,60,66]
	Angiomatoid fibrous histiocytoma	AFH, MIM:612160	[48,56]
*ZMYND15*	Spermatogenic failure 14	SPGF14, MIM:615842	[49]
*TEX15*	Spermatogenic failure 25	SPGF25, MIM:617960	[53,65,71]

**Table 3:** The entry clones obtained from DNASU that were used to generate 3×HA-tagged expression constructs with Gateway cloning.

Gene	Entry Clone
*AATK*	HsCD00821095
*DLGAP4*	HsCD00829035
*IQSEC1*	HsCD00878932
*MCM3*	HsCD00041579
*TFG*	HsCD00041139
*UGP2*	HsCD00513800

## Data Availability

Data are contained within the article or Supplementary Materials. Further data will be made available on request from the authors.

## Supplementary Materials

The following supporting information can be downloaded at: https://www.mdpi.com/article/10.3390/ijms26136459/s1.

## Abbreviations

The following abbreviations are used in this manuscript:

3×FLAG tag	Peptide with the sequence Asp-Tyr-Lys-Asp-His-Asp-Gly-Asp-Tyr-Lys-Asp-His-Asp-Ile-Asp-Tyr-Lys-Asp-Asp-Asp-Asp-Lys
AEBSF	4-(2-Aminoethyl)-benzenesulfonylfluoride hydrochloride
CNS	Central nervous system
EDTA	Ethylenediaminetetraacetic acid
FBS	Fetal bovine serum
HA tag	Peptide with the sequence Tyr-Pro-Tyr-Asp-Val-Pro-Asp-Tyr-Ala
IP	Immunoprecipitation
LC-MS/MS	Liquid chromatography and tandem mass spectrometry
MW	Molecular weight
NIV	Neovascular inflammatory vitreoretinopathy
SDS-PAGE	Denaturing protein gel electrophoresis
SWATH	Sequential Window Acquisition of All Theoretical Mass Spectra
TAILS	Terminal amine isotopic labeling of substrates
WT	wild-type
