# Supplementary material for: The Identification of Proteolytic Substrates of Calpain-5 with N-Terminomics
Source: Int J Mol Sci. 2025 Jul 4;26(13):6459. doi: 10.3390/ijms26136459 (PMC12249614; doi:10.3390/ijms26136459)
Supplement: Supplementary file 1 [file ijms-26-06459-s001.zip › ijms-3701211-supplementary/Figure S1.pdf]

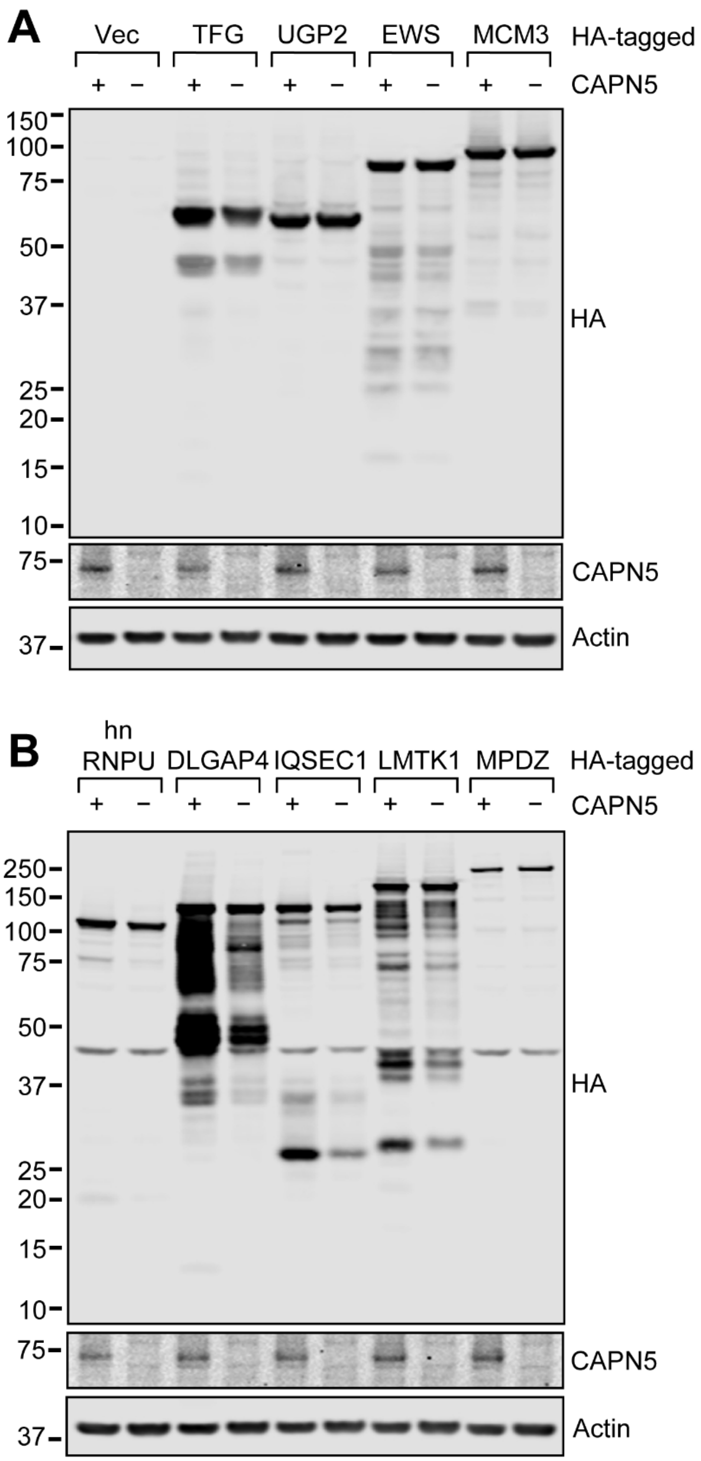

Figure S1. The cellular fragmentation of the 3×HA-tagged putative CAPN5 substrates. Parental or CAPN5$^{-/-}$ SH-SY5Y cells were transfected with expression constructs for the respective 3×HA-tagged putative CAPN5 substrates, followed by lysate preparation, denaturing protein gel electrophoresis and immunoblotting with anti-HA antibody to detect the respective 3×HA-tagged CAPN5 substrate candidates, anti-CAPN5 antibody to confirm the CAPN5 status of the cells and anti-actin antibody as the loading control. The bars indicate molecular weight marker bands (kDa).
