# Supplementary material for: The Identification of Proteolytic Substrates of Calpain-5 with N-Terminomics
Source: Int J Mol Sci. 2025 Jul 4;26(13):6459. doi: 10.3390/ijms26136459 (PMC12249614; doi:10.3390/ijms26136459)
Supplement: Supplementary file 1 [file ijms-26-06459-s001.zip › ijms-3701211-supplementary/Table S4.pdf]

Supplemental Table 4. CAPN5 substrate candidates identified by non-unique TAILS peptides. The entries in bold correspond to the gene within each group that is expressed the highest at the mRNA level in SH-SY5Y cells (The Human Protein Atlas).

*: Potential pseudogene.

UniProt AC	UniProt ID	Gene Name	Protein Name	Cleavage Site
P0C0S8	H2A1_HUMAN	*H2AC11; H2AC13; H2AC15; H2AC16; H2AC17*	Histone H2A type 1	21R A22
Q96QV6	H2A1A_HUMAN	*H2AC1*	Histone H2A type 1-A	21R A22
P04908	H2A1B_HUMAN	*H2AC4; H2AC8*	Histone H2A type 1-B/E	21R A22
Q93077	H2A1C_HUMAN	*H2AC6*	Histone H2A type 1-C	21R A22
P20671	H2A1D_HUMAN	*H2AC7*	Histone H2A type 1-D	21R A22
Q96KK5	H2A1H_HUMAN	*H2AC12*	Histone H2A type 1-H	21R A22
Q99878	H2A1J_HUMAN	*H2AC14*	Histone H2A type 1-J	21R A22
Q6FI13	H2A2A_HUMAN	*H2AC18; H2AC19*	Histone H2A type 2-A	21R A22
Q8IUE6	H2A2B_HUMAN	*H2AC21*	Histone H2A type 2-B	21R A22
Q16777	H2A2C_HUMAN	*H2AC20*	Histone H2A type 2-C	21R A22
Q7L7L0	H2A3_HUMAN	*H2AC25*	Histone H2A type 3	21R A22
Q9BTM1	H2AJ_HUMAN	*H2AJ*	Histone H2A.J	21R A22
Q71UI9	H2AV_HUMAN	*H2AZ2*	Histone H2A.V	23R A24
P16104	H2AX_HUMAN	*H2AX*	Histone H2AX	21R A22
**P0C0S5**	**H2AZ_HUMAN**	***H2AZ1***	**Histone H2A.Z**	**23R A24**
Q58FF7	H90B3_HUMAN	*HSP90AB3P* *	Heat shock protein HSP 90-beta-3	320H G321
P07900	HS90A_HUMAN	*HSP90AA1*	Heat shock protein HSP 90-alpha	386R G387
**P08238**	**HS90B_HUMAN**	***HSP90AB1***	**Heat shock protein HSP 90-beta**	**378R G379**
**A6NHP3**	**SPE2B_HUMAN**	***SPDYE2B***	**Speedy protein E2B**	**110R V111**
P0CI01	SPDE6_HUMAN	*SPDYE6*	Speedy protein E6	110R V111
P04350	TBB4A_HUMAN	*TUBB4A*	Tubulin beta-4A chain	62R A63
**P68371**	**TBB4B_HUMAN**	***TUBB4B***	**Tubulin beta-4B chain**	**62R A63**
Q3ZCM7	TBB8_HUMAN	*TUBB8*	Tubulin beta-8 chain	62R A63
P07477	TRY1_HUMAN	*PRSS1*	Trypsin-1	72R L73
**P07478**	**TRY2_HUMAN**	***PRSS2***	**Trypsin-2**	**72R L73**
Q8NHM4	TRY6_HUMAN	*PRSS3P2* *	Trypsin-6	72R L73
